# Draft Genome Sequence of the Sulfate-Reducing Bacterium *Desulfotomaculum copahuensis* Strain CINDEFI1 Isolated from the Geothermal Copahue System, Neuquén, Argentina

**DOI:** 10.1128/genomeA.00870-16

**Published:** 2016-08-18

**Authors:** Graciana Willis Poratti, Amira Suriaty Yaakop, Chia Sing Chan, M. Sofía Urbieta, Kok-Gan Chan, Robson Ee, Adrian Tan-Guan-Sheng, Kian Mau Goh, Edgardo R. Donati

**Affiliations:** a CINDEFI (CCT, La Plata-CONICET, UNLP), Facultad de Ciencias Exactas, Universidad Nacional de la Plata, La Plata, Argentina; b Faculty of Biosciences and Medical Engineering, Universiti Teknologi Malaysia, Skudai, Johor, Malaysia; c Division of Genetics and Molecular Biology, Institute of Biological Sciences, Faculty of Science, University of Malaya, Kuala Lumpur, Malaysia

## Abstract

*Desulfotomaculum copahuensis* strain CINDEFI1 is a novel spore-forming sulfate-reducing bacterium isolated from the Copahue volcano area, Argentina. Here, we present its draft genome in which we found genes related with the anaerobic respiration of sulfur compounds similar to those present in the Copahue environment.

## GENOME ANNOUNCEMENT

The geothermal Caviahue-Copahue system is a naturally extreme environment located in the northwest of Neuquén province, Argentina and dominated by Copahue volcano. The area is characterized by the presence of acidic, sulfur-rich and high-temperature hot springs that are potential sources of extremophilic microorganisms, with applications in various biotechnological processes, mainly those related with biomining and bioremediation (1, 2). Sulfate-reducing prokaryotes are of special interest since they can be used in heavy metals bioprecipitation processes. Copahue hot springs are enabling environments for the development of such microorganisms, and microorganisms with sulfate-reducing activity have already been detected in the anaerobic zones (3).

To date only three species of this genus have been isolated from terrestrial hot springs (4), the most recent being *Desulfotomaculum copahuensis* strain CINDEFI1 obtained from an acidic sediment from Las Máquinas hot spring (40°C, pH 2) at Copahue volcano. *D. copahuensis* strain CINDEFI1 is a spore-forming and slightly curved rod that grows optimally at 40°C and pH 7.0. It is an obligate anaerobe capable of heterotrophic growth using lactate, pyruvate as well as several sugars as carbon and energy sources. These compounds are incompletely oxidized to acetic acid (5).

Genomic DNA of *D. copahuensis* strain CINDEFI1 was extracted using a Promega Wizard genomic DNA purification kit (Promega) and sequenced using the Illumina HiSeq 2500 platform (Illumina, Inc., San Diego, CA, USA). Genome assembly was performed using CLC Genomics Workbench 7.5 which predicted a genome size of 3,628,787 bp in 375 contigs (largest: 228,398 bp, shortest: 2,008 bp, and *N*_50_ 50,475 bp) with a coverage of 55.76× and 47.7% G+C content. Annotation was performed with NCBI Prokaryotic Genome Annotation Pipeline 2.10 and RAST server (6). A total of 4,063 coding sequences (CDSs), and 59 structural RNAs (51 tRNAs and 8 rRNAs) were predicted. Three percent of the CDSs were classified as hypothetical proteins and 96% as known enzymes.

The genome of *D. copahuensis* strain CINDEFI1 presents genes that might be involved in the metabolic features described previously for this strain. Key enzymes for sulfate reduction, such as sulfate adenylyltransferase (ATP-sulfurilase), adenosine-5′-phosphosulfate (APS) reductase, and dissimilatory sulfite reductase (DSR), were detected (7). Sugar metabolism can be inferred by the presence of all genes of the Embden-Meyerhof-Parnas pathway. Enzymes involved in the fermentation of sugar compounds and a putative pyruvate dehydrogenase complex were also found (8). Incomplete oxidation of organic compounds observed experimentally, can be confirmed by the absence of genes for key enzymes for the TCA cycle. On the other hand, genes codifying enzymes related with energy generation and conservation in Gram-positive sulfate-reducers, including *qmoAB* and *hdrBC* genes, which codify the Qmo complex and the soluble subunits of the heterodisulfide reductase B and C, respectively, were also found (7, 9). All protein sequences showed low e-values with corresponding proteins from *D. thermocisternum, D. nigrificans, D. gibsoniae*, and *D. reducens* in a BLASTp comparison (7, 10).

### Accession number(s).

This whole-genome shotgun project has been deposited at DDBJ/ENA/GenBank under the accession no. LYVF00000000. The version described in this paper is version LYVF01000000.
